# Plasmid-Mediated Transmission of KPC-2 Carbapenemase in *Enterobacteriaceae* in Critically Ill Patients

**DOI:** 10.3389/fmicb.2019.00276

**Published:** 2019-02-19

**Authors:** Christian Schweizer, Peter Bischoff, Jennifer Bender, Axel Kola, Petra Gastmeier, Manfred Hummel, Frank-Rainer Klefisch, Felix Schoenrath, Andre Frühauf, Yvonne Pfeifer

**Affiliations:** ^1^Department of Infection Control/Internal Medicine, Paulinenkrankenhaus, Berlin, Germany; ^2^Department of Infection Control, German Heart Center Berlin, Berlin, Germany; ^3^Institute of Hygiene and Environmental Medicine, Charité – Universitätsmedizin Berlin, Corporate Member of Freie Universität Berlin, Humboldt-Universität zu Berlin and Berlin Institute of Health, Berlin, Germany; ^4^Robert Koch Institute, FG13 Nosocomial Pathogens and Antibiotic Resistances, Wernigerode, Germany; ^5^Department of Cardiothoracic and Vascular Surgery, German Heart Center Berlin, Berlin, Germany; ^6^Partner Site Berlin, DZHK: German Centre for Cardiovascular Research, Berlin, Germany

**Keywords:** multidrug-resistance, *Citrobacter freundii* ST98, *Klebsiella oxytoca* ST29, *Escherichia coli* ST369, IncN, WGS

## Abstract

Carbapenem-resistant *Enterobacteriaceae* (CRE) cause health care-associated infections worldwide, and they are of severe concern due to limited treatment options. We report an outbreak of KPC-2-producing CRE that was caused by horizontal transmission of a promiscuous plasmid across different genera of bacteria and hospitals in Germany. Eleven isolates (8 *Citrobacter freundii*, 2 *Klebsiella oxytoca*, and 1 *Escherichia coli*) were obtained from seven critically ill patients during the six months of the outbreak in 2016. One patient developed a CRE infection while the other six patients were CRE-colonized. Three patients died in the course of the hospital stay. Six of the seven patients carried the same *C. freundii* clone; one *K. oxytoca* clone was found in two patients, and one patient carried *E. coli* and *C. freundii*. Molecular analysis confirmed the presence of a conjugative*, bla*_KPC-2_-carrying 70 kb-IncN plasmid in *C. freundii* and *E. coli* and an 80 kb-IncN plasmid in *K. oxytoca.* All transconjugants harbored either the 70 or 80 kb plasmid with *bla*_KPC-2,_ embedded within transposon variant Tn*4401g*. Whole genome sequencing and downstream bioinformatics analyses of all plasmid sequences showed an almost perfect match when compared to a *bla*_KPC-2_-carrying plasmid of a large outbreak in another German hospital two years earlier. Differences in plasmid sizes and open reading frames point to the presence of inserted mobile genetic elements. There are few outbreak reports worldwide on the transmission of *bla*_KPC-2_-carrying plasmids across different bacterial genera. Our data suggest a regional and supraregional spread of *bla*_KPC-2_-carrying IncN-plasmids harboring the Tn*4401g* isoform in Germany.

## Introduction

“*Klebsiella pneumoniae carbapenemase*” (KPC) was first described in carbapenem-resistant *K. pneumoniae* isolates from the United States in the 1990s. According to the NCBI database more than 30 different KPC variants are known today but KPC-2 and KPC-3 are the most prevalent enzymes worldwide (Tzouvelekis et al., 2012; Mathers et al., 2015). Multilocus sequence typing (MLST) analyses have confirmed the worldwide emergence of the successful clonal group CG258 of *K. pneumoniae*, which includes different sequence types (STs), e.g., ST258, ST512, ST11, ST340, and ST437. KPC-2 or KPC-3-producing *K. pneumoniae-*ST258 are spread worldwide, and over the last 10 years the number of infections has increased dramatically in some countries, for instance the United States, Israel, Greece, and Italy (Mathers et al., 2015; Wyres and Holt, 2016). The *bla*_KPC_ gene is embedded in transposon Tn*4401*, which is located on plasmids of different sizes and replicon type, or is integrated into the bacterial chromosome (Mathers et al., 2017). As a result of its plasmid location, *bla*_KPC_ can be transferred to species other than *K. pneumoniae*, e.g., *Enterobacter* spp., *Citrobacter* spp., or *E. coli;* interspecies transmission during outbreaks has been reported (Mathers et al., 2011, 2017; Tzouvelekis et al., 2012).

Plasmids of the incompatibility group N (IncN) are prevalent in *Enterobacteriaceae* and are known to carry various resistance determinants including extended-spectrum β-lactamases (ESBLs) and carbapenemases like KPC. The 119 IncN plasmids currently deposited in the plasmid MLST database^1^ represent the high plasticity of this plasmid type. Of special interest are IncN plasmids that harbor *bla*_KPC-2_ and the β-lactamase gene *bla*_TEM-1_ in the truncated transposon isoform Tn*4401g*. These plasmids appear to be very “promiscuous” and have been described in different *Enterobacteriaceae* species in Israel and Germany (Chmelnitsky et al., 2014; Yao et al., 2014). A reported outbreak with KPC-2-producing *Enterobacteriaceae* (including *Citrobacter* spp., *Klebsiella oxytoca, Enterobacter* spp., *E. coli*) in Germany in 2014 left 132 patients rectally colonized but without clinical symptoms. The kitchen in the hospital was identified as the probable source of the outbreak after detection of KPC-2 producers (*C. freundii and K. oxytoca*) in different foods (Robert Koch-Institut, 2014). The present study analyzed KPC-2-producing isolates of different species from patients in two other German hospitals for the presence of plasmids as vehicles for transmission of carbapenem resistance.

## Materials and Methods

### Microbiological and Molecular Analysis

Between February 2016 and December 2017, a total of 13 carbapenem-resistant isolates (8 *C. freundii*, 2 *K. oxytoca*, and 3 *E. coli*) were obtained from seven patients hospitalized in two different hospitals in Berlin, Germany. Species identification and determination of antibiotic susceptibilities was performed using an automated system (VITEK 2, AST-N248, bioMérieux, Nürthingen, Germany). The presence of carbapenemase genes (*bla*_NDM-like_, *bla*_OXA-48-like_, *bla*_V IM-like_, *bla*_IMP-like_, and *bla*_KPC-like_) and further β-lactamase genes (*bla*_TEM-like_, *bla*_SHV -like_, *bla*_CTX-M-like_, *bla*_OXA-1-like_, *bla*_OXA-2-like_, *bla*_OXA-9-like_, *bla*_OXA-10-like_, *bla*_CMY_, and *bla*_DHA_) was confirmed by PCR as previously described (Gröbner et al., 2009; Pfeifer et al., 2011). Screening for plasmid-mediated quinolone resistance (PMQR) genes [*qnrA/B/S; aac(6’)Ib-cr*] was additionally performed using primers published in other studies (Park et al., 2006; Pfeifer et al., 2009). The genetic environment of *bla*_KPC_ was analyzed using previously described primers AM07/AM08 (Mathers et al., 2011) and primers TEMg 5′-AGGCAACTATGGATGAACGA-3′ and KPCg 5′-AGAGACAAGACAGCAGAACTAGAC-3′, designed by the National Reference Laboratory for multidrug-resistant Gram-negative bacteria in Bochum, Germany, for rapid identification of the Tn*4401g* isoform (Chmelnitsky et al., 2014).

In addition, the genetic relationship of the isolates was analyzed by pulsed-field gel electrophoresis (PFGE) using XbaI-restricted whole genomic DNA. Macrorestriction patterns were interpreted based on the criteria of Tenover et al. (1995). *E. coli* isolates were assigned to the four main phylogenetic groups (A, B1, B2, and D) and clonal lineage O25b:H4-ST131 using PCR-based assays (Clermont et al., 2000; Blanco et al., 2009).

Transfer of β-lactam resistance was tested for all isolates in broth mating assays using a sodium azide-resistant *E. coli* J53 Azi^r^ recipient (Clowes and Rowley, 1954). Transconjugants were selected on Luria-Bertani agar plates with 200 mg/L sodium azide and 50 mg/L ampicillin. Plasmid content and the plasmid size of clinical isolates and transconjugants were determined by means of S1-nuclease restriction and PFGE as described by Barton et al. (1995). PCR-based replicon typing (“PBRT KIT,” DIATHEVA, Fano, Italy) was performed using plasmid DNA of transconjugants (Carattoli et al., 2005). For comparison of plasmid restriction patterns, EcoRI restriction was performed after plasmid DNA isolation from transconjugants using the Qiagen Plasmid Mini Kit.

### Whole Genome Sequencing and Data Analysis

After performance and evaluation of PFGE-based bacterial strain typing and conjugation assays four isolates (2 *C. freundii*, 1 *E. coli*, and 1 *K. oxytoca*) and their respective KPC-2-positive transconjugants were selected for WGS analysis (Table 1). These eight isolates were cultivated in Brain Heart Infusion (BHI) broth. DNA was extracted from overnight cultures using the MagAttract Kit (Qiagen) and the DNeasy Blood and Tissue Kit (Qiagen) following the manufacturer’s instructions. A Qubit dsDNA HS Assay Kit (Invitrogen) was used for DNA quantification. Sequencing libraries were prepared by applying the Nextera XT DNA Library Prep Kit (Illumina). They were sequenced on an Illumina Miseq using a v3 chemistry (2 × 300 bp) as described in the manufacturer’s protocol. The quality of fastq data obtained was assessed and *de novo* assembly was performed by the in-house developed pipelines QCumber 2.0.0^2^ and batch Assembly 2.1.2. The latter implements the assembler a5-miseq or SPAdes, respectively (Coil et al., 2015)^3^.

**Table 1:** Molecular characteristics of KPC-2 producing *Enterobacteriaceae* isolates.

Isolate no.	RKI-Nr.	Patient no.	Species	Date of isolation	Material	β-lactamase genes	PMQR^3^ genes	PFGE type	Sequence Type^4^	*bla*_KPC-2_-carrying plasmid
And 11164^1^	604-16	#1	*C. freundii*	26.02.2016	TS	*bla*_KPC-2_, *_bla_*_TEM-1_, *bla*_OXA-1_	*aac(6’)Ib-cr*	C-1	ST98	70kb, IncN pST15
And 11165	605-16	#2	*C. freundii*	29.02.2016	Rectal swab	*bla*_KPC-2_, *_bla_*_TEM-1_, *bla*_OXA-1_	*aac(6’)Ib-cr*	C-1a	n.a.	70kb, IncN pST15
RK190946K1	730-17	#2	*E. coli*	Dec 2017^2^	Rectal swab	*bla*_KPC-2_, *_bla_*_TEM-1_, *bla*_OXA-1_	*aac(6’)Ib-cr*	E-2 (B2)^5^	n.a.	n.d.
RK190946K2	731-17	#2	*E. coli*	Dec 2017^2^	Rectal swab	*bla*_KPC-2_, *_bla_*_TEM-1_, *bla*_OXA-1_	*aac(6’)Ib-cr*	E-3 (A)^5^	n.a.	70kb, IncN pST15
And 11166	606-16	#3	*C. freundii*	11.03.2016	Rectal swab	*bla*_KPC-2_, *_bla_*_TEM-1_, *bla*_OXA-1_	*aac(6’)Ib-cr*	C-1b	n.a.	70kb, IncN pST15
And 11167^1^	607-16	#3	*E. coli*	09.04.2016	Rectal swab	*bla*_KPC-2_, *_bla_*_TEM-1_, *bla*_OXA-1_	*aac(6’)Ib-cr*	E-1 (A)^5^	ST369	70kb, IncN pST15
And 11138	608-16	#4	*C. freundii*	16.03.2016	Urine	*bla*_KPC-2_, *_bla_*_TEM-1_, *bla*_OXA-1_	*aac(6’)Ib-cr*	C-1c	n.a.	70kb, IncN pST15
And 11137^1^	609-16	#4	*K. oxytoca*	16.03.2016	Urine	*bla*_KPC-2_, *_bla_*_TEM-1_, *bla*_OXA-1_, *bla*_OXY -like_	*aac(6’)Ib-cr; qnrB2*	K-1	ST29	80kb, IncN pST15
And 11261	752-16	#5	*C. freundii*	29.04.2016	TS	*bla*_KPC-2_, *_bla_*_TEM-1_, *bla*_OXA-1_	*aac(6’)Ib-cr; qnrB2*	C1d	n.a.	70kb, IncN pST15
And 11298	611-16	#6	*C. freundii*	05.05.2016	Throat swab	*bla*_KPC-2_, *_bla_*_TEM-1_, *bla*_OXA-1_	*aac(6’)Ib-cr; qnrB2*	C1e	n.a.	70kb, IncN pST15
RK 171170-2	827-16	#7	*C. freundii*	29.07.2016	Rectal swab	*bla*_KPC-2_, *_bla_*_TEM-1_, *bla*_OXA-1_	*aac(6’)Ib-cr; qnrB2*	C-1f	n.a.	70kb, IncN pST15
RK 171170-3^1^	828-16-1	#7	*C. freundii*	29.07.2016	Rectal swab	*bla*_KPC-2_, *_bla_*_TEM-1_, *bla*_OXA-1_	*aac(6’)Ib-cr; qnrB2*	C-1f	ST98	70kb, IncN pST15
RK 171170-1	829-16	#7	*K. oxytoca*	29.07.2016	Rectal swab	*bla*_KPC-2_, *_bla_*_TEM-1_, *bla*_OXA-1_, *bla*_OXY -like_	*aac(6’)Ib-cr; qnrB2*	K-1a	n.a.	80kb, IncN pST15


*^1^four clinical isolates and the respective transconjugant were selected for WGS analysis^2^new admission of patient no. 2 in 2017; ^3^PMQR plasmid-mediated quinolone resistance; ^4^multilocus sequence types extracted from WGS data using webtool MLST 1.8/cgMLST; ^5^E. coli phylogenetic group according to Clermont et al. (2000); n.a., not analyzed (isolate was not selected for WGS analysis); n.d., not determined (broth mate conjugation failed); TS, tracheal secretion; RKI, Robert Koch Institute.*

Contigs, obtained after *de novo* assembly, were submitted to the CGE Finder Series (Centre for Genomic Epidemiology, Technical University of Denmark (DTU)^4^, pubmlst.org (University of Oxford, Great Britain) and to the SeqSphere+ software suite (Ridom GmbH, Muenster, Germany). Different analysis tools (MLST and pMLST from pubmlst.org, cgMLST, or ResFinder 3.0) were applied to extract the multilocus ST as described by (Wirth et al., 2006) the plasmid multilocus sequence type (pST), and information on genes that mediate resistance to β-lactams, fluoroquinolones and other antibiotics (Larsen et al., 2012; Zankari et al., 2012; Carattoli et al., 2014; Joensen et al., 2014, 2015).

The BLAST Ring Image Generator (BRIG) (Alikhan et al., 2011) was used to align all assembled reads of the strains sequenced to the *bla*_KPC-2_-carrying *C. freundii* plasmid pCF8698 [accession no. LN610760, 54,036 bp (Yao et al., 2014)]. In addition, mapping of selected transconjugant reads to *C. freundii* pCF8698 or to the transposon Tn*4401g* carrying *bla*_KPC-2_ (Chmelnitsky et al., 2014) was performed using Geneious 10.0.5.

### Sequence Data/Accession Numbers

Raw read data was assigned SRA Accession No. SRP153806.

### Ethics Statement

No informed consent or ethical approval was required since all isolates were generated and analyzed as part of microbiological diagnostics (therapeutic purposes) and/or infection prevention and control requirements and measures.

The outbreak investigation was conducted in accordance with article 25, section 1 of the German Infection Protection Act of 2001.

## Results

### Clinical Setting and Outbreak Description

**FIGURE 1:**
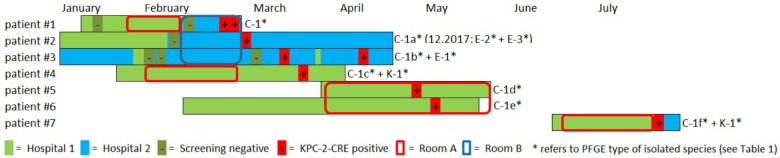
Overview and description of the outbreak in 2016. Colored bars represent the patient’s stay in hospital 1 (light green) and hospital 2 (light blue). Circles indicate the stay in a certain ICU room (red = room A, blue = room B). Negative and positive microbiological results (screening or clinical specimen) for each patient is indicated with (–) and (+), respectively. A carbapenem-resistant *C. freundii* (PFGE type C1) was isolated from both urine and tracheobronchial secretion in the index patient (#1) in hospital 2. Both contact patients (#2 and #3) in the 3-bed-room (room B) were positive for carbapenem-resistant *C. freundii* over the course of two weeks (rectal screening). Both a KPC-2-producing *C. freundii* (rectal) and a KPC-2-producing *K. oxytoca* (urine) were isolated from patient #4 on a peripheral ward in hospital 1. Patient #1 and patient #4 had shared a 2-bed-room in the ICU of hospital 1 five weeks earlier. Nine weeks after patients #1 and #4 had left, KPC-2-producing *C. freundii* were detected in other patients in this room. For patient #5, a positive result was obtained from tracheobronchial secretion and the contact patient #6 had a rectal and pharyngeal colonization. Patient #7 was admitted to the same ICU 2-bed-room in hospital 1 about one month later and occupied the room for about five weeks. Only after discharge and during admission screening in hospital 2 both a KPC-2-producing *C. freundii* and *K. oxytoca* (both rectal) were isolated. In patient #2 two KPC-2 producing *E. coli* morphotypes were isolated upon re-admission 20 months later.

Between February and July 2016, cases, all cardiosurgical patients with major comorbidities, were identified in two separate intensive care units (ICUs) and one peripheral ward all spread across two different hospitals in the same city. Unveiling this epidemiological link was possible due to close cooperation of the local infection prevention and control teams. A carbapenem-resistant *C. freundii* was isolated from both urine and tracheobronchial secretion in the index patient (#1) in hospital 2 (Figure 1). Both contact patients (#2 and #3) in the 3-bed room were found to be positive for carbapenem-resistant *C. freundii* by rectal screening over the course of two weeks. Prior to the initial pathogen detection, the index patient had been hospitalized for about six weeks, four of those in hospital 1. Notably, none of the previous screenings (on average two per patient) had yielded a carbapenem-resistant strain in any of these three patients. About four weeks after the contact patients tested positive, a carbapenem-resistant *E. coli*, along with *C. freundii*, was found by rectal screening of patient #3.

The National Reference Laboratory for multidrug-resistant Gram-negative bacteria in Bochum, Germany, identified carbapenemase gene *bla*_KPC-2_ in all the isolates as the cause of resistance. Seemingly independent of this situation, both a KPC-2-producing *C. freundii* (rectal swab) and a KPC-2-producing *K. oxytoca* (urine) were isolated from another patient (#4) on a peripheral ward in hospital 1. However, epidemiological investigation revealed that patient #1 and patient #4 had shared a 2-bed room in the ICU of hospital 1 five weeks earlier. Nine weeks after patients #1 and #4 had left, KPC-2-producing *C. freundii* were detected in other patients in this room. In patient #5, a positive result was obtained from a tracheobronchial secretion and the contact patient (#6) exhibited rectal and pharyngeal colonization. The room was thoroughly disinfected and all materials there were discarded, as far as possible.

Patient #7 was admitted to the same ICU 2-bed-room in hospital 1 about one month later and occupied the room for about five weeks. Only after discharge and upon admission screening (rectal swabs) in hospital 2, both KPC-2-producing *C. freundii* and *K. oxytoca* were isolated. Three of the patients (#1, #5, and #6) died as a result of their underlying diseases. One patient developed a CRE infection (#1), while the other six patients remained colonized during their stay and at discharge. Information on the previous travel history of the patients was not available. All carbapenem-resistant isolates identified were sent to the Robert Koch Institute (RKI) for further analysis.

Outbreak investigation identified stay in the same ICU room as the most obvious epidemiological link (room A+B; Figure 1). Moreover, one particular ICU room was found to be the common source for CRE acquisition for five patients who had all had stays in that room at separate times over the course of a six months period (patients #1,#4,#5,#6,#7, room A; Figure 1). Environmental investigation for CRE was not carried out during the patient stays. After the discharge of the last patient, the ICU ward in hospital 1 was completely renovated and no new cases have occurred ever since. After 20 months, one of the CRE carrying patients (patient #2) was admitted to the hospital again when screening revealed colonization with two carbapenem-resistant *E. coli* morphotypes.

### Microbiological and Molecular Analysis

Between February and July 2016, 11 carbapenem-resistant isolates (8 *C. freundii*, 2 *K. oxytoca*, and 1 *E. coli*) were obtained from seven patients in two different hospitals. The isolates were resistant to imipenem, meropenem, piperacillin/tazobactam, cefotaxime, ceftazidime, aztreonam, ciprofloxacin, moxifloxacin, gentamicin, tobramycin, and trimethoprim/sulfamethoxazole. Susceptibility to amikacin, colistin, tigecyline, and fosfomycin was confirmed in all isolates.

XbaI-macrorestriction patterns of *C. freundii* isolated from six patients in the period February–May 2016 varied in 1–4 bands, which confirmed the presence of the same clone (PFGE types C1a-e, Table 1). Two *C. freundii* isolates of patient #7 isolated at the end of July 2016 showed a difference in 6–7 bands in their macrorestriction pattern compared to the previous isolates. According to the criteria of Tenover et al. (1995) and Mathers et al. (2011), this difference is to be interpreted as showing a possible relation or no relation of these isolates. Because of the epidemiological link of the patients and WGS-based assignment of both isolates to ST98, we decide to assign both isolates to the same clone as all the other *C. freundii* isolates. Furthermore, PFGE analysis confirmed the presence of one *K. oxytoca* clone in two patients, and three *E. coli* clones were identified (patients #2 and #3; Supplementary Figure S1). Finally, utilizing WGS and the programs MLST and cgMLST of pubmlst.org and SeqSphere+, respectively, both *C. freundii* isolates were typed ST98, the *K. oxytoca* clone was typed ST29 and the *E. coli* clone as a member of ST369 and complex type CT789 (Table 1).

PCR and sequencing confirmed the presence of the carbapenemase gene *bla*_KPC-2_ in all 13 isolates (Table 1). This gene showed a unique genetic environment, the Tn*4401g* transposon variant with *bla*_TEM-1_ next to *bla*_KPC-2_, as described previously in *Enterobacteriaceae* isolates from Israel and in outbreak isolates from Germany in 2014 (Chmelnitsky et al., 2014; Robert Koch-Institut, 2014; Yao et al., 2014). Broth mating successfully transferred the *bla*_KPC-2_-carrying plasmids of approx. 70–80 kb and IncN group. All transconjugants harbored only one plasmid which were either 70 kb (*E. coli* and *C. freundii* donor isolates) or 80 kb (*K. oxytoca* donor) plasmids (Table 1 and Supplementary Figure S2). All transconjugants were positive for β-lactamase genes *bla*_KPC-2,_ *bla*_OXA-1,_ and *bla*_TEM-1,_ and PMQR gene *aac(6’)Ib-cr.* PMQR gene *qnrB2* was additionally present in transconjugants and their respective clinical isolates (*C. freundii* from three patients and the two *K. oxytoca* isolates (Table 1).

**FIGURE 2:**
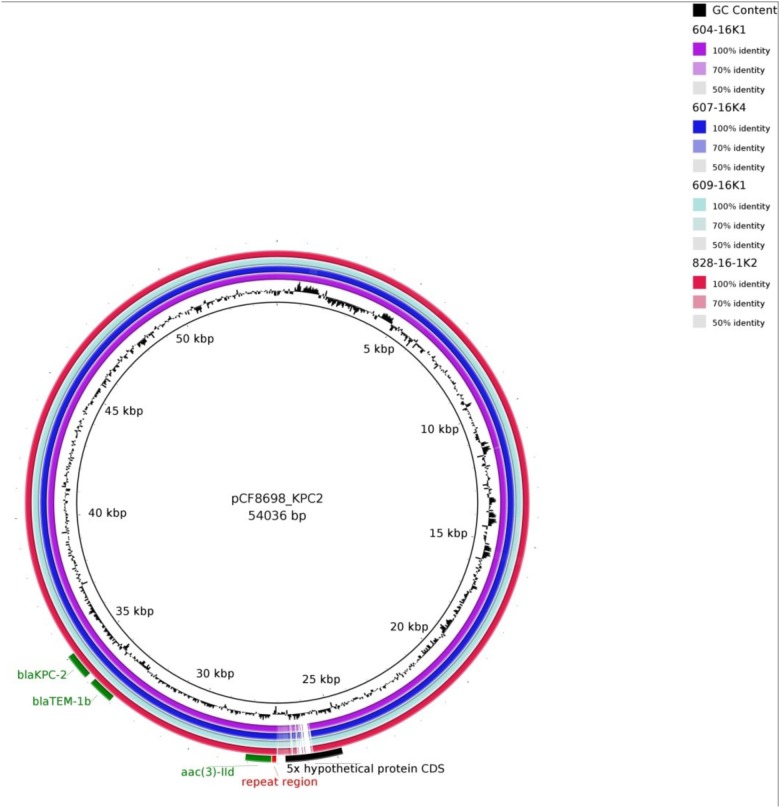
BLAST Ring Image Generator analysis of plasmid sequences. Plasmid sequences of four transconjugants were compared with plasmid pCF8698_KPC2 (Yao et al., 2014). violett, 604-16K1 *E. coli* K12J53 *bla*_KPC-2_, *bla*_TEM-1_, *bla*_OXA-1_, *aac(6’)Ib-cr* (patient #1); dark blue, 607-16K4 *E. coli* K12J53 *bla*_KPC-2_, *bla*_TEM-1_, *bla*_OXA-1_, *aac(6’)Ib-cr* (patient #3); light blue, 609-16K1 *E. coli* K12J53 *bla*_KPC-2_, *bla*_TEM-1_, *bla*_OXA-1_, *aac(6’)Ib-cr, qnrB2* (patient #4); red, 828-16-1K2 *E. coli* K12J53 *bla*_KPC-2_, *bla*_TEM-1_, *bla*_OXA-1_, *aac(6’)Ib-cr, qnrB2* (patient #7); important resistance genes are labeled in green.

Simple EcoR1-restriction of the 70 and 80 kb plasmids revealed similar restriction patterns (Supplementary Figure S3). Use of WGS and the tool pMLST, identified the IncN plasmids of all transconjugants as pST15. The pMLST database^5^ contains only three entries of IncN-pST15 plasmids, identified in *E. coli* and *K. pneumoniae* in Israel in 2005–2006 (Goren et al., 2010; Leavitt et al., 2010). A BRIG analysis of all plasmid sequences showed an almost perfect match with the *bla*_KPC-2_-carrying 54 kb-IncN plasmid pCF8698_KPC2 (accession no. LN610760). This plasmid was isolated from *C. freundii* from a KPC-mediated outbreak in a German hospital in 2014 (Yao et al., 2014). Minor deviations in coverage and gaps from the sequence of pCF8698_KPC2 were noted in a region comprising five hypothetical proteins (Figure 2). In accordance with differences in plasmid sizes in pCF8698 and the ones investigated here, it was presumed that insertion of mobile genetic elements (MGEs) or deletion of certain plasmid fragments had occurred. Therefore, paired-end reads were aligned to the reference plasmid pCF8698_KPC2, followed by a MAUVE alignment of contigs of interest. MGEs possibly inserted in the region of the five hypothetical proteins could not be determined. However, we detected a 777 bp insertion in gene *secA* in all isolates of the present outbreak that encodes an *IS*1 family transposase (BLASTn results, data not shown).

Another 13.202 bp insertion in the gene *umuC* was present only in the 80 kb *bla*_KPC-2_-carrying plasmid in *K. oxytoca*. BLASTn analysis of the inserted sequence revealed regions homologous to plasmid pKPN-edaa (accession no. NZ_CP026398.1) from *K. pneumoniae* isolated in an ICU piping system of an United States hospital in 2017 (Weingarten et al., 2018). The sequence encodes hypothetical proteins, a membrane anchor protein, a Tat signal sequence, an MFS transporter, and the nickel/cobalt efflux protein RcnA. The size of the inserted sequence corresponds consistently to the size difference of the *K. oxytoca* plasmid demonstrated by S1-nuclease PFGE (Supplementary Figure S2).

It must be noted that transconjugants TC 604-16K1 of *C. freundii* and TC 607-16K4 of *E. coli* demonstrate a 3^rd^ insertion site in a hypothetical protein located around nucleotide position 29.460. The fragment revealed homologies to regions from plasmids of various gram-negative pathogens and comprises a DDE transposase and genes belonging to arsenical efflux (data not shown).

The transconjugants harboured *bla*_KPC_, further β-lactamase and PMQR genes and in addition a number of other antibiotic resistance genes. Using ResFinder with a threshold of 90% identity and a minimum length of 60%, acquired resistance genes that confer resistance to rifampicin, phenicols, aminoglycosides, trimethoprim, sulfonamide, and macrolides were extracted, e.g., catB3, arr-3, strA, dfrA18, sul1, and mph(A) (Supplementary Table S1).

## Discussion

In Germany, the first outbreak with KPC-2-producing *K. pneumoniae* was detected in 2007–2008 followed by another large outbreak in 2010/13 (Wendt et al., 2010; Lübbert et al., 2014). In both outbreaks admission of patients previously hospitalized in Greece were identified as possible index patients. Transmission of *bla*_KPC-2_ to other species, e.g., *E. coli*, was only detected in a single case in the outbreak 2007–2008. Subsequently performed analyses in the RKI revealed another genetic environment of this gene (*istB* gene next to *bla*_KPC-2_, Mathers et al., 2011) which is in contrast to the isolates of the present study. This MGE was integrated into a conjugative 100 kb plasmid. Unlike the worldwide spread of KPC-producing *K. pneumoniae* clones, e.g., ST258, there are several outbreak reports on the transmission of promiscuous *bla*_KPC-2_-carrying plasmids across different bacterial species and genera (Mathers et al., 2011; Chmelnitsky et al., 2014; Robert Koch-Institut, 2014; Huang et al., 2018; Kukla et al., 2018; Li et al., 2018).

The location of *bla*_KPC-2_ on conjugative IncN plasmids in different bacterial species from patients and environmental samples has been described worldwide, and *bla*_KPC-2_ was embedded primarily in the Tn*4401b* isoform (Pereira et al., 2015; Eilertson et al., 2017; Nascimento et al., 2017; Weingarten et al., 2018). The transfer of these different plasmids between adapted species (e.g., *Klebsiella* spp., *Citrobacter* spp., and *Enterobacter* spp.) in biofilms of the aquatic environment in hospitals (e.g., sinks, pipes) suggests that these are an important reservoir for resistance genes (Weingarten et al., 2018). In the present study *bla*_KPC-2_ was embedded in the Tn*4401g* isoform in an IncN-pST15 plasmid that had been described only in *Enterobacteriaceae* isolates from Israel and Germany (Chmelnitsky et al., 2014; Yao et al., 2014). Consistent with the isolates of the outbreak in Hesse, Germany, in 2014, the isolates in present outbreak were primarily of species *C. freundii and K. oxytoca.* Furthermore, patients with co-colonization indicate possible events of plasmid transfer to commensal *E. coli* in the gut or in the hospital environment. According to the MLST database, the *E. coli*-ST369 identified in this study seems to be widespread. ST369 was identified previously in poultry, food and some clinical samples in Germany, the Netherlands, the United States and Japan^6^ (Ozawa et al., 2010).

WGS analyses showed a high degree of similarity of the *bla*_KPC-2_-carrying IncN-plasmids of the 2016 outbreak in Berlin to these of the 2014 outbreak in Hesse, Germany. Nevertheless, the sizes of the *bla*_KPC-2_-carrying plasmids varied, which is caused primarily by insertions of various MGEs. Because short read sequencing is limited with respect to sequence reconstruction, we were not able to identify all inserted MGEs or to complete the entire plasmid sequences. This is a limitation of the present study.

In summary, we have confirmed another multispecies-outbreak caused by a *bla*_KPC-2_-carrying Tn*4401g* transposon on IncN plasmids that occurred in two German hospitals two years after a large outbreak in a hospital located at a distance of 500 km. In the present outbreak, the hospital environment seemed to have played a particular role as a source of KPC-producing *C. freundii*. Over a course of six months five patients with consecutive, separate stays in the same hospital room acquired the same CRE clones. Although the surfaces and objects in the room had been thoroughly disinfected multiple times, the unknown source was only eliminated when a planned renovation of the ward was carried out. Transfer of colonized patients and contamination of the aquatic environment with KPC-producing bacteria might have facilitated the regional and supraregional spread of *bla*_KPC-2_-carrying plasmids.

## Author Contributions

CS and PB carried out the epidemiological investigations. AK, PG, MH, F-RK, and FS supervised and supported this work. JB, AF, and YP planned and carried out the experiments. CS, PB, JB, and YP drafted the manuscript and designed the figures. All authors contributed to the interpretation of the results, provided critical feedback and helped to finalize the manuscript.

## Conflict of Interest Statement

The authors declare that the research was conducted in the absence of any commercial or financial relationships that could be construed as a potential conflict of interest.

## Footnotes

1 http://pubmlst.org/plasmid/ (July 18, 2016)

2 https://gitlab.com/RKIBioinformaticsPipelines/QCumber

3 http://cab.spbu.ru/software/spades/

4 https://cge.cbs.dtu.dk/services/

5 https://pubmlst.org/plasmid/

6 http://enterobase.warwick.ac.uk/species/ecoli/search_strains
