# Supplementary material for: Plasmid-Mediated Transmission of KPC-2 Carbapenemase in Enterobacteriaceae in Critically Ill Patients
Source: Front Microbiol. 2019 Feb 19;10:276. doi: 10.3389/fmicb.2019.00276 (PMC6390000; doi:10.3389/fmicb.2019.00276)
Supplement: Supplementary file 2 [file Presentation_1.PPTX]

## Slide 1
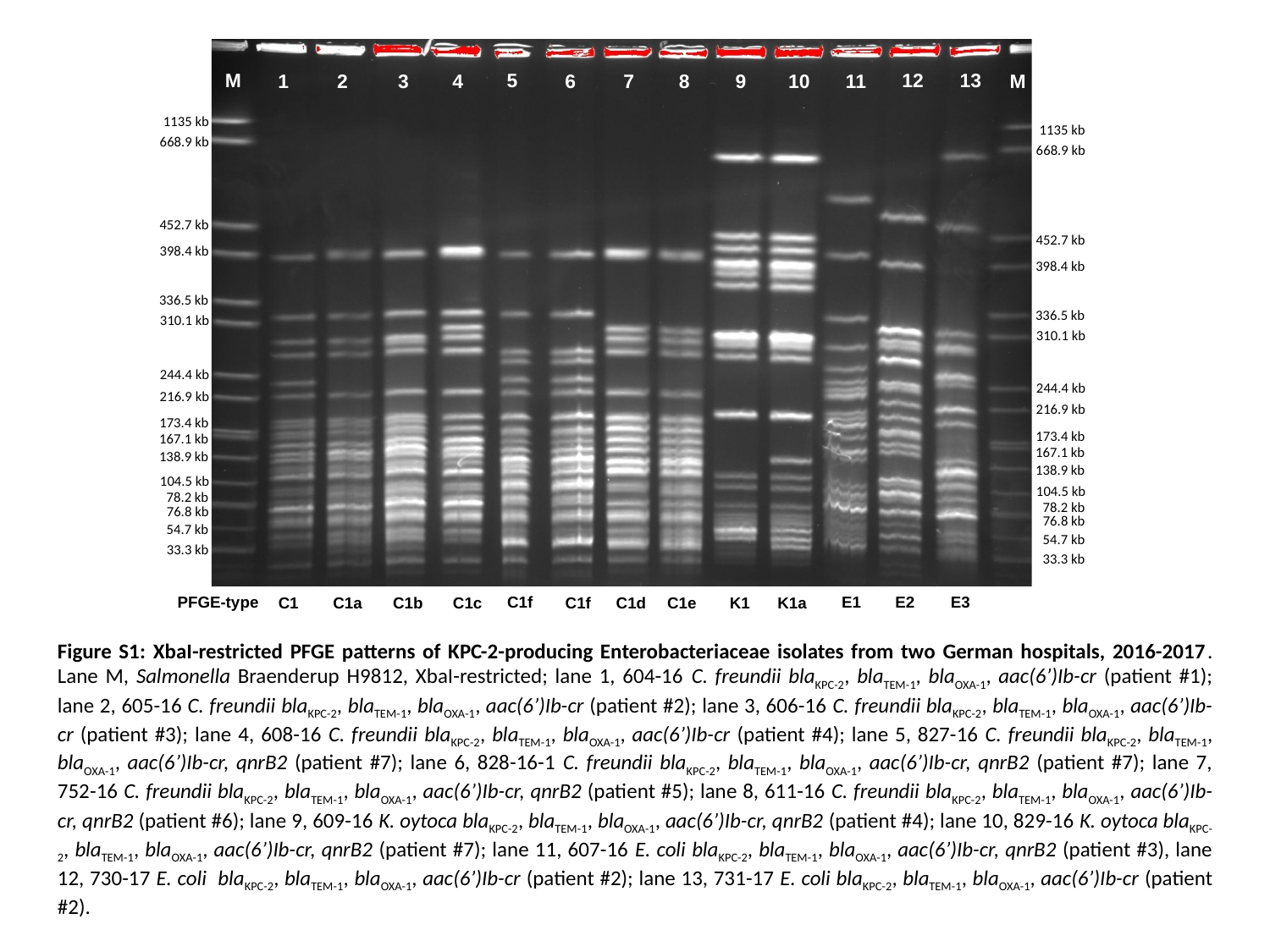

12
13
5
M
11
M
4
6
8
10
9
1
2
3
7
1135 kb
1135 kb
668.9 kb
668.9 kb
452.7 kb
452.7 kb
398.4 kb
398.4 kb
336.5 kb
336.5 kb
310.1 kb
310.1 kb
244.4 kb
244.4 kb
216.9 kb
216.9 kb
173.4 kb
173.4 kb
167.1 kb
167.1 kb
138.9 kb
138.9 kb
104.5 kb
104.5 kb
78.2 kb
78.2 kb
76.8 kb
76.8 kb
54.7 kb
54.7 kb
33.3 kb
33.3 kb
E2
E3
C1f
PFGE-type
E1
C1c
C1f
C1e
K1a
K1
C1
C1a
C1b
C1d
Figure S1: XbaI-restricted PFGE patterns of KPC-2-producing Enterobacteriaceae isolates from two German hospitals, 2016-2017. Lane M, Salmonella Braenderup H9812, XbaI-restricted; lane 1, 604-16 C. freundii blaKPC-2, blaTEM-1, blaOXA-1, aac(6’)Ib-cr (patient #1); lane 2, 605-16 C. freundii blaKPC-2, blaTEM-1, blaOXA-1, aac(6’)Ib-cr (patient #2); lane 3, 606-16 C. freundii blaKPC-2, blaTEM-1, blaOXA-1, aac(6’)Ib-cr (patient #3); lane 4, 608-16 C. freundii blaKPC-2, blaTEM-1, blaOXA-1, aac(6’)Ib-cr (patient #4); lane 5, 827-16 C. freundii blaKPC-2, blaTEM-1, blaOXA-1, aac(6’)Ib-cr, qnrB2 (patient #7); lane 6, 828-16-1 C. freundii blaKPC-2, blaTEM-1, blaOXA-1, aac(6’)Ib-cr, qnrB2 (patient #7); lane 7, 752-16 C. freundii blaKPC-2, blaTEM-1, blaOXA-1, aac(6’)Ib-cr, qnrB2 (patient #5); lane 8, 611-16 C. freundii blaKPC-2, blaTEM-1, blaOXA-1, aac(6’)Ib-cr, qnrB2 (patient #6); lane 9, 609-16 K. oytoca blaKPC-2, blaTEM-1, blaOXA-1, aac(6’)Ib-cr, qnrB2 (patient #4); lane 10, 829-16 K. oytoca blaKPC-2, blaTEM-1, blaOXA-1, aac(6’)Ib-cr, qnrB2 (patient #7); lane 11, 607-16 E. coli blaKPC-2, blaTEM-1, blaOXA-1, aac(6’)Ib-cr, qnrB2 (patient #3), lane 12, 730-17 E. coli blaKPC-2, blaTEM-1, blaOXA-1, aac(6’)Ib-cr (patient #2); lane 13, 731-17 E. coli blaKPC-2, blaTEM-1, blaOXA-1, aac(6’)Ib-cr (patient #2).

## Slide 2
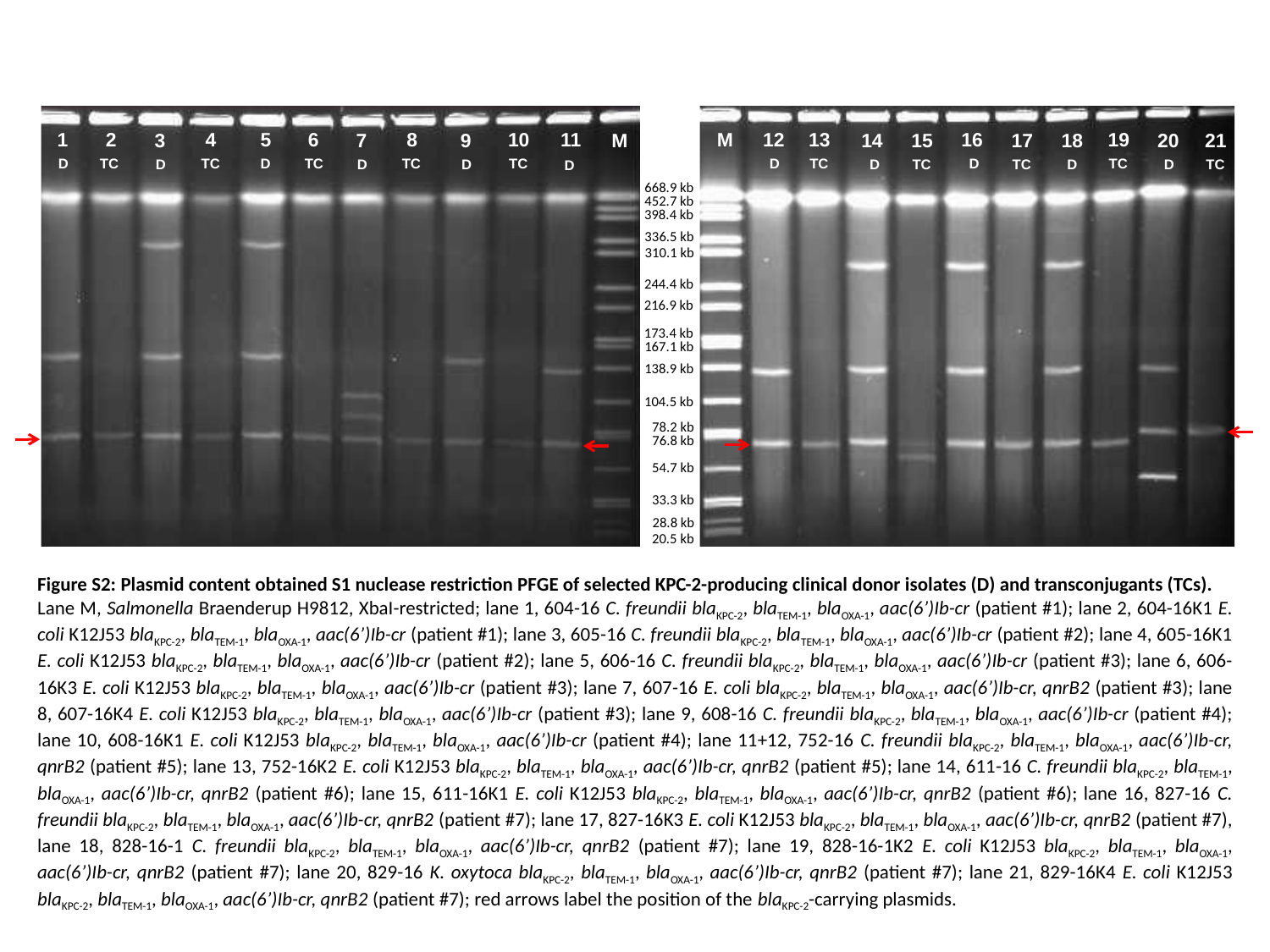

5
16
2
19
8
11
10
1
6
13
12
4
M
21
3
M
18
20
15
14
9
17
7
D
D
TC
TC
TC
TC
D
TC
TC
D
TC
TC
D
D
D
TC
D
D
TC
D
D
668.9 kb
452.7 kb
398.4 kb
336.5 kb
310.1 kb
244.4 kb
216.9 kb
173.4 kb
167.1 kb
138.9 kb
104.5 kb
78.2 kb
76.8 kb
54.7 kb
33.3 kb
28.8 kb
20.5 kb
Figure S2: Plasmid content obtained S1 nuclease restriction PFGE of selected KPC-2-producing clinical donor isolates (D) and transconjugants (TCs).
Lane M, Salmonella Braenderup H9812, XbaI-restricted; lane 1, 604-16 C. freundii blaKPC-2, blaTEM-1, blaOXA-1, aac(6’)Ib-cr (patient #1); lane 2, 604-16K1 E. coli K12J53 blaKPC-2, blaTEM-1, blaOXA-1, aac(6’)Ib-cr (patient #1); lane 3, 605-16 C. freundii blaKPC-2, blaTEM-1, blaOXA-1, aac(6’)Ib-cr (patient #2); lane 4, 605-16K1 E. coli K12J53 blaKPC-2, blaTEM-1, blaOXA-1, aac(6’)Ib-cr (patient #2); lane 5, 606-16 C. freundii blaKPC-2, blaTEM-1, blaOXA-1, aac(6’)Ib-cr (patient #3); lane 6, 606-16K3 E. coli K12J53 blaKPC-2, blaTEM-1, blaOXA-1, aac(6’)Ib-cr (patient #3); lane 7, 607-16 E. coli blaKPC-2, blaTEM-1, blaOXA-1, aac(6’)Ib-cr, qnrB2 (patient #3); lane 8, 607-16K4 E. coli K12J53 blaKPC-2, blaTEM-1, blaOXA-1, aac(6’)Ib-cr (patient #3); lane 9, 608-16 C. freundii blaKPC-2, blaTEM-1, blaOXA-1, aac(6’)Ib-cr (patient #4); lane 10, 608-16K1 E. coli K12J53 blaKPC-2, blaTEM-1, blaOXA-1, aac(6’)Ib-cr (patient #4); lane 11+12, 752-16 C. freundii blaKPC-2, blaTEM-1, blaOXA-1, aac(6’)Ib-cr, qnrB2 (patient #5); lane 13, 752-16K2 E. coli K12J53 blaKPC-2, blaTEM-1, blaOXA-1, aac(6’)Ib-cr, qnrB2 (patient #5); lane 14, 611-16 C. freundii blaKPC-2, blaTEM-1, blaOXA-1, aac(6’)Ib-cr, qnrB2 (patient #6); lane 15, 611-16K1 E. coli K12J53 blaKPC-2, blaTEM-1, blaOXA-1, aac(6’)Ib-cr, qnrB2 (patient #6); lane 16, 827-16 C. freundii blaKPC-2, blaTEM-1, blaOXA-1, aac(6’)Ib-cr, qnrB2 (patient #7); lane 17, 827-16K3 E. coli K12J53 blaKPC-2, blaTEM-1, blaOXA-1, aac(6’)Ib-cr, qnrB2 (patient #7), lane 18, 828-16-1 C. freundii blaKPC-2, blaTEM-1, blaOXA-1, aac(6’)Ib-cr, qnrB2 (patient #7); lane 19, 828-16-1K2 E. coli K12J53 blaKPC-2, blaTEM-1, blaOXA-1, aac(6’)Ib-cr, qnrB2 (patient #7); lane 20, 829-16 K. oxytoca blaKPC-2, blaTEM-1, blaOXA-1, aac(6’)Ib-cr, qnrB2 (patient #7); lane 21, 829-16K4 E. coli K12J53 blaKPC-2, blaTEM-1, blaOXA-1, aac(6’)Ib-cr, qnrB2 (patient #7); red arrows label the position of the blaKPC-2-carrying plasmids.

## Slide 3
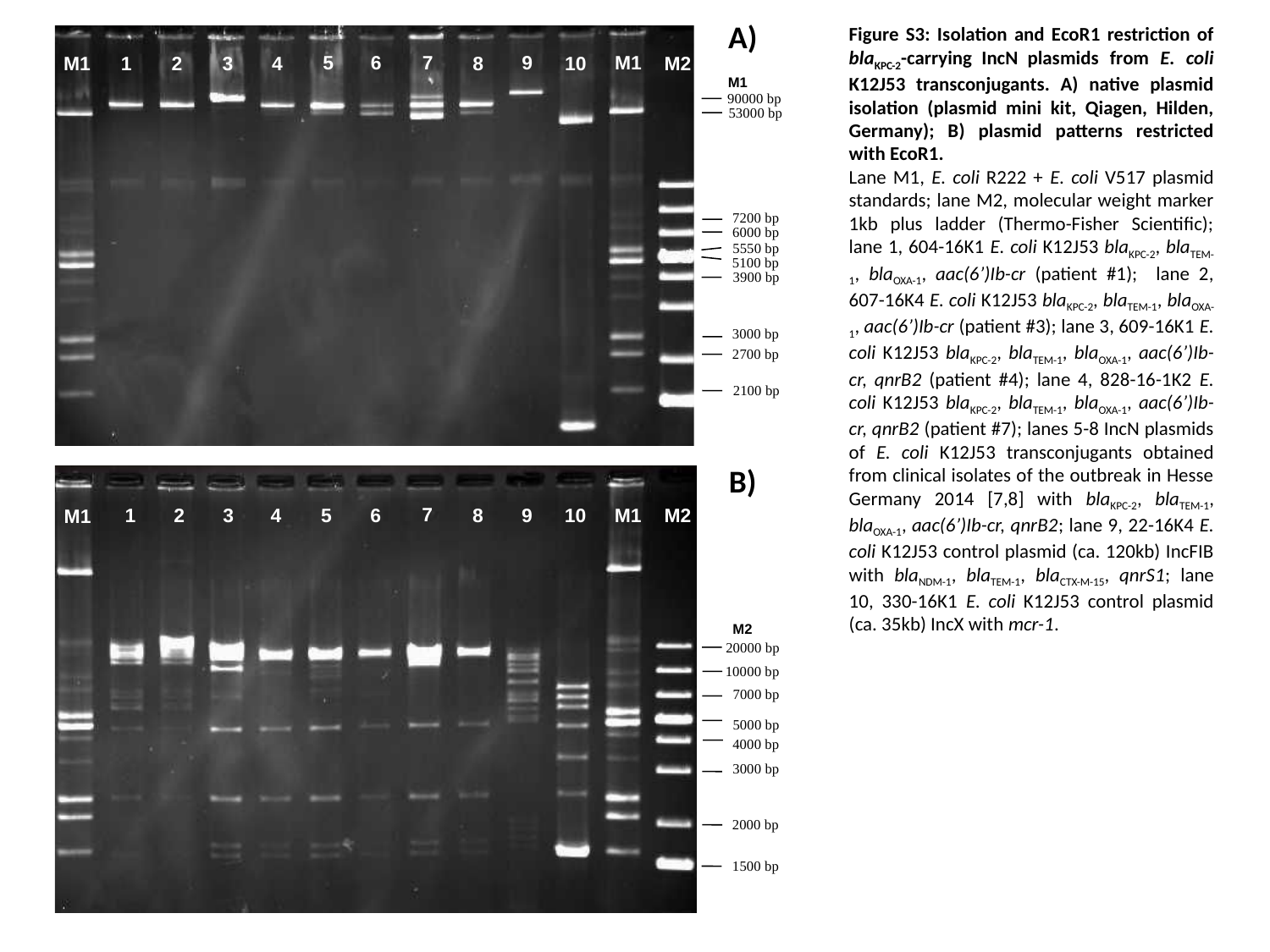

A)
Figure S3: Isolation and EcoR1 restriction of blaKPC-2-carrying IncN plasmids from E. coli K12J53 transconjugants. A) native plasmid isolation (plasmid mini kit, Qiagen, Hilden, Germany); B) plasmid patterns restricted with EcoR1.
Lane M1, E. coli R222 + E. coli V517 plasmid standards; lane M2, molecular weight marker 1kb plus ladder (Thermo-Fisher Scientific); lane 1, 604-16K1 E. coli K12J53 blaKPC-2, blaTEM-1, blaOXA-1, aac(6’)Ib-cr (patient #1); lane 2, 607-16K4 E. coli K12J53 blaKPC-2, blaTEM-1, blaOXA-1, aac(6’)Ib-cr (patient #3); lane 3, 609-16K1 E. coli K12J53 blaKPC-2, blaTEM-1, blaOXA-1, aac(6’)Ib-cr, qnrB2 (patient #4); lane 4, 828-16-1K2 E. coli K12J53 blaKPC-2, blaTEM-1, blaOXA-1, aac(6’)Ib-cr, qnrB2 (patient #7); lanes 5-8 IncN plasmids of E. coli K12J53 transconjugants obtained from clinical isolates of the outbreak in Hesse Germany 2014 [7,8] with blaKPC-2, blaTEM-1, blaOXA-1, aac(6’)Ib-cr, qnrB2; lane 9, 22-16K4 E. coli K12J53 control plasmid (ca. 120kb) IncFIB with blaNDM-1, blaTEM-1, blaCTX-M-15, qnrS1; lane 10, 330-16K1 E. coli K12J53 control plasmid (ca. 35kb) IncX with mcr-1.
7
M1
5
6
9
M1
10
M2
1
2
3
8
4
M1
90000 bp
53000 bp
7200 bp
6000 bp
5550 bp
5100 bp
3900 bp
3000 bp
2700 bp
2100 bp
B)
7
M2
1
2
3
8
4
M1
5
6
9
10
M1
M2
20000 bp
10000 bp
7000 bp
5000 bp
4000 bp
3000 bp
2000 bp
1500 bp
